# Safeguarding Children Subjected to Violence in the Family: Child-Centered Risk Assessments

**DOI:** 10.3390/ijerph192113779

**Published:** 2022-10-23

**Authors:** Maria Eriksson, Anders G. Broberg, Ole Hultmann, Emma Chawinga, Ulf Axberg

**Affiliations:** 1 Department of Social Sciences, Marie Cederschiöld University, SE 11628 Stockholm, Sweden; 2 Department of Psychology, University of Gothenburg, SE 40530 Gothenburg, Sweden; 3 Faculty of Social Sciences, VID Specialized University, NO 0370 Oslo, Norway

**Keywords:** child abuse, child agency, child development, risk assessment, violence

## Abstract

Assessing risk, planning for safety and security, and aiding recovery for children subjected to violence in a family setting is a complex process. The aim of the article is to synthesize the current research literature about risks for children subjected to violence in the family and outline an empirical base for a holistic and practically usable model of risk assessments placing the individual child at the center. Such assessments need to recognize four different areas of risk: (1) child safety, i.e., known risk factors for severe and dangerous violence aimed at both adults and children and how they play out in the individual case; (2) the child’s response in situations with violence; (3) the child’s perspective, especially fear and feelings of powerlessness in situations with violence; (4) developmental risks, e.g., instability in the child’s situation and care arrangements, lack of a carer/parent as a “secure base” and “safe haven”, the child developing difficulties due to the violence (e.g., PTSD), problems in parents’ caring capacities in relation to a child with experiences of, and reactions to, violence, and lack of opportunities for the child to make sense of, and create meaning in relation to, experiences of violence. In addition to the four areas of risk, the article emphasizes the importance of assessing the need for immediate intervention and safety planning in the current situation as regards safety, the child’s responses, the child’s perspectives, and long-term developmental risks.

## 1. Introduction

A significant minority of children experience intimate partner violence (IPV) while growing up. For example, in the United Kingdom, 12 per cent of children under 11 years old, 17.5 per cent of children aged 11–17, and 23.7 per cent of 18–24-year-olds report having been exposed to violence between adults in their homes during childhood [1]. In several countries in the global north, it is estimated that approximately five per cent of children experience violence regularly [2,3,4]. In other parts of the world, the figures may be higher. Since the early 1980s, both research and practice with women subjected to IPV have shown that IPV is an issue of direct concern for children. Most children in these families have been exposed to the violence [5,6], which can be defined as psychological abuse of the child, and in Sweden it has been a criminal act since 2021 [7]. In addition, there is an overlap between IPV and direct abuse of the children in the family, especially in IPV cases of men’s violence against women [8,9,10,11]. In most cases, it is the perpetrator of IPV, typically the father or stepfather, who is abusive also towards the child. However, sometimes the abused parent, typically the mother or stepmother, may be using violence or harsh discipline as well. As regards child maltreatment outside of the context of IPV, studies has tended to be biased, as all but a very small proportion of studies include only mothers in their samples [11].

Children’s exposure to violence in the family is often long term and becomes part of their everyday life. Therefore, it affects all aspects of their relationships with parents and siblings and also with agemates and adults outside the family. Exactly how a specific child is affected in her/his development is determined by protective and vulnerability factors in the child, but also by risk and protective factors in the environment. Risk assessment therefore must focus both on how dangerous the current situation is and on long-term risk for child development.

There is now a substantial body of research documenting the detrimental effects of exposure to psychological, physical, and sexual violence in a family setting for children’s health and well-being in both the short and long term [12,13]. However, research also shows that the situation and consequences of exposure to IPV for the individual child will vary [14,15]. Thus, careful consideration of the needs of the particular child is necessary for any agency intervening in the family [16,17,18]. Another way to describe the overwhelming experiences from exposure to IPV and abuse for children, is by using the concept of complex trauma [19], which is classified as a separate psychiatric condition for adults in the ICD 11 manual. For children, the concept of developmental trauma disorder is used [20]. The concept refers to chronic exposure to interpersonal violence and abandonment from caregivers undermining or reversing developmental attainments. Children exposed to IPV and/or abuse can also be diagnosed with complex trauma if the violence is repetitive, prolonged, and pervasive, but especially young children are more often diagnosed with developmental trauma. Reactions in children are behavioral, emotional, cognitive, and social and they may be diagnosed with multiple psychiatric diagnoses.

A key issue to safeguard and support children exposed to IPV is the assessment of risk, as the level and type of risk need to be considered when deciding on the type of intervention. Children’s exposure to violence in their family is often present during a substantial period of their childhood: a chronic condition, not just a single discernible event. How children are affected in their development is determined by protective and vulnerability factors in the individual child, but also depend on such factors in the environment. There is an urgent need for knowledge development and practice improvement when it comes to risk assessment. Studies indicate that cases referred to child welfare services due to violence may be left without intervention, and then often reoccur [21]. Some children are even abused while being in contact with child welfare services [12,22]. 

In the literature on risk assessment in the context of IPV, commentators have pointed out that the more widely used risk assessment instruments or methods are adapted to violence perpetrators found in a criminal justice context, rather than child protection or health care, e.g., [23]. Principles from investigative interviews [24] with suspects are usable in the risk assessment with parents who have perpetrated IPV or abused children, but they are not suitable for interviews with victims of violence in the family. As both adults and children may be subjected to violence, and there may be several perpetrators in the same family, assessing risk is a complex matter. The complexity of these cases where both adults and children are at risk has led some to argue that we need step-by-step models for risk assessment where case workers draw on different instruments and methods to assess the risk to partners and children, and then integrate the results into an overall conclusion [23,25]. This conclusion is in line with reviews of different methods of risk assessment concluding that although some studies indicate that actuarial methods, that is, methods that have been developed from empirical research about risk and predictive factors, are generally more accurate than unstructured methods [26], there are no instruments or methods that have shown to be superior to others [27] or cover the risks for both adults and children in a comprehensive way.

Another shortcoming of the current approaches to risk assessment is the lack of recognition of children’s agency and perspectives [28]. The knowledge development on children and IPV has for a long time been dominated by quantitative studies primarily using mothers, but not their children, as informants, and drawing on perspectives from developmental psychology and social medicine [14,29,30,31]. However, in the last decades, there has been a growing body of studies—in the Nordic countries and the United Kingdom especially—of children and IPV that have included children as informants and explored their agency and views: see, e.g., [32,33,34,35,36]. This development is in line with the influence from the “new” sociology or social studies of children and childhood that has gained ground within all fields of research on children since the early 1990s [37,38,39]. 

So far, knowledge on children’s agency and perspectives has not been integrated into the research on risk assessment to any greater extent. Risk assessment and IPV tends to focus on immediate danger and perpetrator dangerousness. Although crucial, a focus on the perpetrator of IPV is not enough in a child-centered risk assessment model taking children’s developmental needs and agency into account. Long-term or developmental risks for children must be assessed in parallel with the assessment of immediate danger and safety. Deficits in parenting linked to perpetration or victimization can be a serious long-term risk factor for children’s ability to develop and adjust to new challenges and tasks in life. In addition, in the case of a trauma, contact that is experienced as unsafe by the child may be re-traumatizing for the child and have long-term deleterious effects on the child’s development. A holistic risk assessment model thus needs to include the child’s sense of security, and the assessment will have to include long-term risk and how different care and contact arrangements can aid the child’s recovery. Furthermore, children are not ”passive” victims of situations with violence; instead, they may attempt to intervene and manage these situations, sometimes in ways that put them at risk for harm [40,41]. Thus, children’s own actions must also be considered. Finally, risk assessments need to include several sources of information, including both parents, children, and other sources. A model for collecting information, compiling information, and making a decision must thus inevitably build on different bodies of knowledge, childhood sociology, developmental psychology, investigative interviewing, violence dynamics, etc. As risk is a dynamic phenomenon, risk and protective factors will have to be considered in relation to each other. Finally, both immediate and long-term risks need to be assessed.

The aim of the article is to synthesize the current research literature about risks for children subjected to violence in the family and outline an empirical base for holistic risk assessments placing the individual child at the center. In addition, to aid the development of professional practice in this area, the synthesis needs to be focused enough to be possible to implement in everyday child welfare practice. It is argued that assessments need to recognize at least four different components of risk: (1) child safety; (2) the child’s responses in situations with violence; (3) the child’s perspective; and (4) developmental risks. In addition, an assessment of the need for immediate intervention and safety planning in the current situation must consider all of these areas: safety, the child’s responses, the child’s perspectives, and long-term developmental risks. 

## 2. Theoretical Framework, Materials, and Methods

The theoretical framework underpinning the article is a synthesis of childhood sociology, developmental psychology, and developmental psychopathology, viewing children as both social actors with their own views and competencies, and as developing individuals in need of adult protection, care, and guidance [42,43]. A key concept is children as social actors, which highlights children’s competence and participation in research as well as their social life [37]. Such a conceptualization of children does, of course, not exclude a developmental perspective or the possibility that children need protection and support from adults [28]. Children are thus regarded on the one hand as subjects, and as objects on the other, and it is with this dual approach to children that the current research literature on children and IPV has been reviewed.

An explorative and narrative review [44] of the research literature was conducted, analyzing and summarizing several different bodies of literature, to identify and highlight key areas for a holistic and child-centered model of risk assessment. Existing systematic reviews and meta-analyses as well as primary studies and textbooks have been considered, depending on the state of knowledge in the particular sub-field. There are, for example, a number of meta-analyses of evidence for the psychological effects of exposure to IPV, but no systematic reviews of research about children’s responses in situations with violence. Additionally, literature reviews and meta-analyses tend to focus on research conducted in the U.S. and other parts of the English-speaking world, and it is not a given that these results are directly transferable to other contexts. There are, for example, considerable differences between countries when it comes to attitudes and practices regarding corporeal punishment, which is criminalized in some parts of the world, but not in others [4].

The review of research literature and synthesis of knowledge was carried out by a multidisciplinary research team representing psychology, sociology, and social work. The context was a larger project of developing a model for child-centered risk assessment in collaboration with practitioners within child welfare services, as described elsewhere. As the literature is vast—for example, at the time of writing, a simple search in a database such as PubMed on ‘domestic violence’, ‘children’, and ‘risk assessment’ renders over 2000 articles—a pragmatic approach was necessary to cover the broad range of research relevant to the everyday risk assessment practice within child welfare services, and further, within the constraints given by the development project, to be able to outline an agenda for child-centered risk assessments that would be grounded in research yet usable in practice and possible to test empirically in future studies. 

The starting point for the exploration of the research literature was the authors’ previous work in the field of children and IPV and pre-existing reviews of research on children and IPV, e.g., [29,30,36] and risk assessment, respectively, e.g., [26,27]. The first step of analysis was to identify areas of risk necessary to consider in a holistic model for risk assessment. Four areas were identified and are outlined in the Results section. The next step was then to review previous research within these areas more thoroughly. Within each area, searches in both major (e.g., PsychInfo, PubMed, Scopus, Web of Science) and more specialized databases (e.g., Social Work Abstracts) were conducted. Search terms were adapted to the specific area (e.g., ‘child agency’, ‘risk assessment’, and ‘child welfare’ for the area about the child’s response). Review articles and meta-analyses were reviewed, and when deemed appropriate original studies included in the reviews were followed-up upon as well. Relevant original articles and textbooks within each area were reviewed. It is primarily research published in the last 20 years that has been included; however, the review also draws on some older texts when relevant (e.g., theoretical texts). The authors strived to identify the factors with the strongest support within the literature, as consensus was searched for within and across several different bodies of literature. 

The review resulted in an empirical base for a holistic and comprehensive risk assessment model to be used in everyday practice, that places the individual child at the center and is informed by research on violence and risk, developmental psychopathology, and childhood sociology. The assessment model is based on four areas of assessment: child safety, the child´s response, the child’s perspective, and developmental risks. The empirical research results were grouped into these four areas of risk for several reasons. Considering the topic of the review and practice development, the child’s safety was a given area, and as the review concerns children, so were developmental risks. Regarding research results that highlight children’s own views and actions, they were divided into two separate areas. Firstly, the research makes it clear that there is no given link between what children do and how they feel and view their situation; thus, each of these areas of risk needs to be assessed in its own right. Secondly, child welfare work can be dominated by adult perspectives and a view of children as objects for adult care and control. Highlighting two separate child agency-focused areas to assess may enable a stronger focus on children as subjects and actors in child welfare assessment processes.

A selection of just over a hundred studies and other texts were identified as the most relevant considering the aim of the review. They are presented in the Results Section. Some texts are included in several tables as they are relevant to the assessment of several areas of risk. The methodological approach of the review means that parts of the research literature on children and IPV deemed less central to risk assessment have not been included, with the most substantial parts being studies on psychosocial interventions to help children to work through experiences of violence and studies on legal and welfare system responses to children exposed to IPV.

## 3. Results: Areas of Risk

### 3.1. Child Safety

Violence in a family context can be very dangerous, sometimes lethal, for both adults and children. Known risk factors for serious and lethal violence against adults and/or children in a family context such as mental health problems, addiction, and separation or divorce, all need to be considered in the overall assessment. Key aspects that should be considered are summarized in Table 1 and below.

#### 3.1.1. Serious and/or Escalating Violence

The assessment should include an estimation of the level of seriousness of psychological, physical, and sexual violence, and whether the violence has escalated, as both seriousness and escalations are indications of a high level of risk for further violence against both partners and children [50,58,63]. 

#### 3.1.2. Perpetrator Situation

The assessment needs to consider factors in the social situation of the perpetrator such as being out of work, engagement in other criminal activities, access to weapons, social isolation, recent separation or divorce, and a close environment of violence and other forms of abuse [49,50,59,61,63]. 

#### 3.1.3. Perpetrator Characteristics

The assessment needs to consider perpetrator characteristics indicating a high level of risk, such as mental health problems (e.g., anti-social problems, PTSD), explosive temper, ongoing addiction, plans for violence or suicide, misogyny, jealously and a stalking behavior [49,50,59,61,63].

#### 3.1.4. Low Level of Mentalization/Reflective Functioning

The assessment needs to consider the perpetrator’s level of mentalization/reflective functioning [55] when it comes to the use of violence and the consequences for the victims [69]. A limited ability to recognize the partner and/or child as an individual with independent thoughts, needs, and intentions and a lack of tolerance for the partner’s or child’s emotional expressions (e.g., anger or fear) may enable further violence. According to a meta-analysis by Stith et al. [11], the quality of the parent–child relationship is related to physical abuse, and parent perception of the child as a problem is an important risk factor for physical abuse.

#### 3.1.5. Victim Vulnerability

The assessment needs to consider the vulnerability of the adult victim, such as a lack of social stability, financial dependence on the perpetrator, social isolation, resistance against receiving help, a high level of fear, mental health problems, or ongoing addiction, as all of these factors may undermine the victim’s opportunities for protection and support [45,46,50]. 

#### 3.1.6. Signs of Child Abuse

In terms of serious violence against children, visible signs of violence and/or neglect such as bruises or fractures, child behavior problems, somatic symptoms (e.g., pain in the stomach or head), a neglected appearance, and/or absence from school must be carefully considered, as these can all indicate serious and possibly lethal violence against children [11,52,60,64].

#### 3.1.7. Parental Characteristics

Many cases of serious and lethal violence against children occur in cases already known to authorities; thus, previous reports need to be considered carefully. Both parental characteristics (e.g., parent ager/hyper-reactivity, history of abuse, or intellectual disability, [68]) and family factors (conflict level, family cohesion) are important risk factors for child physical abuse [11].

#### 3.1.8. Family Characteristics

There are also some factors associated with the family and social relations that need to be considered when it comes to violence targeting the child, such as social isolation; a positive attitude towards, and habitual use of, corporeal punishment or hash discipline [52]; a high level of interparental conflict; stress and financial difficulties [54,62,66]; and a tendency to blame the child for being abused [11,56].

#### 3.1.9. Child Characteristics

Age is important to consider since younger children are at a higher risk of being maltreated repeatedly, with more serious consequences and death [65,67]. Another aspect to consider is the increased risk for maltreatment and exposure to IPV among children with disabilities [51,53].

### 3.2. The Child’s Response

Research on children’s strategies in relation to violence at home shows that it is important to explore children’s responses in situations with violence, both as part of the assessment of the level of danger and safety, and as the need for interventions teaching children less dangerous ways of responding to situations with violence (e.g., withdrawing, seeking help, etc.). Key aspects that should be considered are summarized in Table 2 and below.

#### 3.2.1. Intervening in Situations with Violence

Situations where the child is intervening physically to stop the violence are particularly dangerous as regards violence targeting the child directly, as well as when children/youth take part in violent acts against any family member [5,34,74].

#### 3.2.2. Intervening by Using Violence

Instances when the child is using violence her or himself to stop the perpetrator from using violence are also situations of risk for the child [5,71].

#### 3.2.3. Protecting Siblings

Assessors need to recognize that children may protect their sibling just as much as they would protect a parent and pay careful attention to indications that a child takes responsibility for helping or protecting a sibling against abuse or witnessing IPV [72,73,75]. 

### 3.3. The Child’s Perspective

Assessments including the perspective of the individual child can be regarded as an expression of the dual approach to children and a holistic perspective where adult protection and care is combined with the child’s right to express her or his view in all matters that concern the child. In addition, research indicates that there is a moderate to low agreement between parent and child reports on the violence the child has experienced [70,76]. Key aspects that should be considered are summarized in Table 3 and below.

#### 3.3.1. Feelings of Fear

Feeling safe and living without fear is a right for children. It is also key to development and recovery after experiences of violence, as continued feelings of fear and unsafe contacts with the perpetrator can undermine support and treatment interventions [22,77]. It is especially important to consider the child’s perspective on fear and safety if the child continues to live with or has regular contact with the perpetrator, as perpetrators may continue violence or engage in post-separation stalking, thus undermining the child’s sense of safety and security [78,79].

#### 3.3.2. Not Knowing What to Do/Feelings of Helplessness

Another key aspect to consider is if the child does not know what to do in situations with violence in order to be and feel safer. Feelings of helplessness and a perceived lack of space for action in the situation [80] adds to the stress experienced by the child and may contribute to the problems some children develop in the aftermath of violence, such as symptoms of traumatic stress.

#### 3.3.3. The Child’s Perspective Is Unknown/or: The Child’s Voice Is Silenced

Some children do not get the opportunity to have a say about their own feelings, experiences, and views, due to, for example, young age, individual difficulties in communicating, or a particularly stressful or pressured situation. Since knowledge about the perspective of the particular child is key to the assessment of risk for that child, a lack of opportunities to express herself, or her perspective, should in itself be considered a risk factor. Research shows that one of the most common effects of exposure to violence for children is feelings of fear [80]; thus, assessors cannot presume that the child feels safe enough, when not knowing that this is the case. The tendency of parents to underestimate what children have been exposed to, and the effects of the exposure [70,76] should also be considered.

### 3.4. Developmental Risks

As outlined in the introduction to the article, there is now a substantial body of research documenting the detrimental effects of exposure to psychological, physical, and sexual violence in a family setting on children’s development. 

Key aspects that should be considered when it comes to risks associated with children’s development in the aftermath of experiences of violence are summarized in Table 4 and below.

#### 3.4.1. Unstable Social Situation

A child’s development, health, and well-being are aided by a stable social situation when growing up [43,110]. Unfortunately, children exposed to violence often suffer an unstable situation due to the violence; they may experience separation and divorce or moving several times to a shelter or other different forms of accommodation. The reason for this can, for many abused and separated mothers, be the violence itself or a lack of financial resources. An unstable housing situation may also be associated with repeated disruptions of school, the loss of social networks and friends, etc., all of which could otherwise have been sources of support to children’s recovery. To reduce risk, the child’s social situation needs to be as stable as possible. 

#### 3.4.2. Unstable Care Arrangements

Stability is a key issue also when it comes to care arrangements. Repeated legal disputes regarding the child may undermine the stability of the care arrangements, and it is a risk for the child’s development if decisions on child custody/parental responsibility, contact/access, and residence do not take the child’s need for stability in the aftermath of violence into account. 

#### 3.4.3. Lack of a “Safe Haven” and “Secure Base”

Exposure to violence may negatively affect the child’s attachment to both the perpetrating and abused parent [97] and contribute to long-term mental health problems of different kinds [91,93,103]. Assessors of risk need to pay attention to signs that the child lacks trust in each parent’s ability or willingness to act as a “safe haven” and “secure base”, i.e., be there for the child and support the child’s emotional regulation, and comfort and protect the child in situations when the child feels scared, lonely, and so forth. 

#### 3.4.4. Symptoms of Problems due to Violence

Systematic reviews and meta-analyses of the effects of IPV exposure for children show that children may develop behavioral problems, emotional problems, and symptoms of post-traumatic stress [14,31,84,88,107]. The risk for both short-term and long-term problems increases if the violence is serious and the exposure prolonged [111]. In addition, children exposed to IPV are commonly also abused directly, thus being double exposed, which in the research has shown to increase the risk of the child developing various symptoms [18,108]. Assessors need to pay attention to the symptoms of problems developed by the child, and the possible need for support and treatment due to violence exposure. It is also important to consider to what extent parents recognize the child’s issues and need for support and/or treatment. 

#### 3.4.5. Normalizing Violence

Even if the child has not developed visible behavioral or emotional problems or symptoms warranting treatment, assessors need to pay attention to signs that the child has normalized violence or developed attitudes condoning violence. Viewing violence as an acceptable way of responding to your partner in intimate relationships may put the child at risk of experiencing violence as a young adult or adult [89,92]. 

#### 3.4.6. Inadequate Care from the Abusive Parent

Perpetrating violence is in itself an expression of inadequate care, regardless of if the violence is aimed at the child directly or the child’s other parent. Being abusive in a family context disregards responsibility for the whole of the child’s situation and relationships, and violence against a child’s parent/carer and attachment figure undermines the child–abused parent relationship [96]. Another key aspect of inadequate care for a child previously exposed to violence by the perpetrating parent is limited reflective functioning in relation to the violence and a lack of ability to understand the situation from the child’s perspective. In addition, tendencies to minimize the violence or its consequences, externalizing and blaming the other parent or child, are other problematic behaviors when caring for a child who has experienced violence [83].

#### 3.4.7. Inadequate Care from the Victimized Parent

Being an abused parent entails caring for a child in a context of stress [85]. Most studies on abused parents concern mothers with young children, and this body of research indicates that the capacity for care can be affected negatively by being abused; however, many mothers try to provide adequate care also under difficult circumstances [100,101,112]. Research shows that abused mothers are not a homogenous group, as some are affected negatively regarding their capacity for care because of, e.g., depression, PTSD symptoms, etc., in the aftermath of violence, while others are not [81]. There are studies indicating that some mothers’ capacity for care is, on the contrary, positively affected due to an enhanced effort to compensate for the strain that is put upon a child subjected to violence [87,102,106]. 

It is thus important to make careful assessments of the individual parent/carer.

#### 3.4.8. Inadequate Care Alliance between the Parents

When parents share the responsibility for care, the quality of care is associated with the care alliance between the parents [82,98]. An inadequate or lacking care alliance undermines the parents’ ability to collaborate and co-parent the child [86]. The ability to form and uphold a care alliance between the parents always needs to be assessed in relation to the position as the perpetrating or abused parent [86]. A parent who has used violence needs to demonstrate change and a willingness to engage in co-parenting in a constructive way, while the abused parent may need time and support to trust the other parent to share the responsibility for care of the child [90]. 

#### 3.4.9. Limited Opportunities to Make Sense of the Violence

A number of studies indicate that it can be difficult for children to make sense of the violence they experience at home [32,34,60]. When children are not able to understand the violence, it becomes harder for them to judge which situations may escalate into violence, and thus they live in constant fear [33]. Not understanding may also be associated with feelings of guilt, shame, and being responsible [105]. Culturally, violence in the private realm tends to be surrounded by taboo [99], making opportunities for children to share their experiences with others scarce. Therefore, children often need help to make sense of the violence and develop a narrative self in relation to the violence [104]. Assessors need to consider if the child is left to her- or himself to try to understand the situation and previous experiences, and to what extent the parents will talk to the child in such a way that the child is able to form a narrative self that includes the history of violence, or talk about the violence in such a way that the child is developing a problematic understanding of the violence and/or the abused parent (e.g., that the violence did not occur, was justified, etc.).

## 4. Discussion

### 4.1. Assessing Several Areas of Risk 

The analysis and summary of research evidence outlined in the Results section above offer a suggestion as to what to assess in a holistic and child-centered model, in terms of child safety, the child’s responses, the child’s perspective, and developmental risks.

#### 4.1.1. Child Safety

As regards assessing danger, a number of instruments exist, and some have been developed specifically for assessing dangerousness in IPV, for example, the Ontario Domestic Assault Risk Assessment (ODARA), The Domestic Violence Risk Appraisal Guide (DVRAG), the Spousal Assault Risk Assessment Guide (SARA), and the Danger Assessment (DA) [26,27,57]. Instruments like these have been shown to increase the accuracy of assessment, but only marginally. There is a lack of prospective longitudinal studies, studies predicting lethal violence, and studies clearly demonstrating that the instruments have been used as intended. Typically, the predictive validity of instruments used in several different national contexts, e.g., DA and SARA, is moderate [57]. Fewer studies have concerned the level of dangerousness of child abuse [11]. Some studies provide empirical support to structured assessments (e.g., the California Family Risk Assessment, CFRA [113]), while other studies point in a different direction [114].

Even though there is no consensus regarding which instruments or methods to use, and the predictive validity of particular instruments is moderate to low, there is a general agreement on the importance of considering the level of risk, and some risk factors are reoccurring in the research.

#### 4.1.2. The Child’s Response

Children living in families with violence can act to protect themselves, a sibling, or a parent [5,40,41,75]. Without putting blame on children, assessors need to recognize that children may put themselves in harm’s way, as the perpetrator may not consider the needs of the child, or even becoming violent towards a child who is ”interfering” in the situation. Children may be expected by family and kin to act as protection, especially when becoming older [73], and children may even use violence themselves to stop the perpetrator [71]. Although not necessarily successful, sometimes children manage to stop the violence, at least temporarily. However, intervening physically by walking between the perpetrator and victim, and/or trying to grab, push, or hit the perpetrator, etc., puts the child at risk for violence and adds to the level of dangerousness. The literature also demonstrates the importance of recognizing children’s relationships with their siblings, as children may not just intervene to protect adults, but also other children in the family.

#### 4.1.3. The Child’s Perspective

Parents often underestimate the level of violence the child has seen, heard, and in other ways experienced, and adults do not necessarily know how the individual child views the situation or the level of fear from which the child is suffering. Different children in the same family may experience the situation in quite different ways. To gain insight into the child’s perspective on the violence, and the child’s own possibilities to act to feel safer, children need to be able to talk about how they see their situation and express their views on what feels safe enough. Exploring the child’s perspective is key to safeguarding the child’s right to protection. Furthermore, continued experiences of violence and feelings of fear will undermine attempts to aid the child’s recovery.

#### 4.1.4. Developmental Risks

Generally speaking, developmental risks are a matter of a complex interaction between different factors over time: personal vulnerabilities (that the child is born with or develops at an early age), risk factors associated with the family and close environment when growing up (including exposure to violence), and protective factors (such as a supportive environment). When conducting child welfare investigations, case workers assess risk and protective factors at several different levels: the child, family, social situation, etc. [43,95]. In the context of such an overall assessment, there are some aspects that seem to be particularly important to consider in the light of the history of violence. 

A key aspect is the parent’s capacity for care, i.e., a parent’s ability to aid the child in solving developmental tasks, by responding to the child’s shifting needs when growing up, and in the particular environment in which the child and parent find themselves [94]. Sometimes, the concept of parenting is used, but here capacity for care is preferred in order to stress that this is what a child needs from a carer, regardless of if he/she is a parent or other adult. Considering the way experiences of violence cause fear and feelings of unsafety, insecurity, and helplessness, a particularly important aspect is the way a carer works as a “secure base” and “safe haven” for the child, i.e., can provide protection, comfort, and help the child with emotional regulation [97]. The research on children and development also point to the importance of considering the needs of the individual child due to different individual vulnerabilities, experiences, coping strategies, views, etc. among children.

### 4.2. Assessment in A Short-Term and Long-Term Perspective

Individual circumstances or several risk factors taken together may indicate that there is a need for immediate intervention and safety planning to protect the child and prevent further harm. As the level of risk may change rapidly, the need for immediate intervention must be assessed both at first contact and during the investigation or treatment process, against the backdrop of any additional information. The body of knowledge outlined above makes it clear that the assessment of the need for immediate intervention must be holistic and take all of the areas of risk into account.

#### 4.2.1. Child Safety, the Child’s Response, and the Child’s Perspective

The point of a child-centered risk assessment model is that in addition to dangerousness, other aspects of risk also must be considered in relation to a possible need for immediate intervention, to secure children’s rights to protection and opportunities for health and development both in the short term and long term. Immediate intervention and safety planning may be warranted as the level of dangerousness is assessed as high, but also because of the other areas of risk, as when the child tends to intervene physically in situations with violence or uses violence her-/himself, or the child expresses a high level of fear, lacks ways of making her-/himself feel safer in situations of violence, or the perspective of the child concerned is not known. 

#### 4.2.2. Immediate Intervention and Developmental Risks

The assessment of developmental risks does imply a long-term perspective and future-oriented measures. However, developmental risks should be considered also here-and-now and when assessing the need for immediate intervention and safety planning. To safeguard the child who has already experienced violence from further harm, circumstances of the current situation such as repeated violence and prolonged exposure; the child currently showing symptoms of negative effects of the violence, e.g., PTSD, complex trauma reactions, or behavior problems; particular vulnerabilities (e.g., young age or disability); or inadequate care from a parent undermining child safety and security in a situation of crisis, may also warrant immediate intervention.

### 4.3. The Need for Method Development

There is clearly a need for method development as regards the risk assessment when children experience violence in a family context. No existing instruments or methods have been shown to be superior to others [27,47,114] or cover the risks for both adults and children in a comprehensive way [48]. Interviewing an alleged perpetrator of IPV or child abuse in a child welfare investigation requires a different interview strategy compared to interviewing a non-violent/victimized parent or child. Knowledge from investigative interviewing with adults [24] and children [115] can inform method development. In addition, current approaches to risk assessment tend to disregard children’s agency and perspectives [28]. In the new millennium, a growing number of studies on children’s IPV and other forms of violence have been drawing on perspectives from childhood sociology [32,33,34,35,36], and there is now time to integrate this body of knowledge with more developmental perspectives and into research on risk assessment. The areas outlined above can serve as a starting point for such development. 

When doing so, some lessons from more general debates on risk, risk assessment, and child development can be learnt. Firstly, in general, the risk of negative development increases with the number of risk factors present. However, secondly, it needs to be recognized that research about risk and protective factors tends to concern patterns at a group level. Statistically significant associations between risk factors and later difficulties are not necessarily relevant for the situation or development of an individual child. This means, thirdly, that it is not a given that a particular child will suffer further violence or develop problems or difficulties in spite of a number of risk factors identified. Protective factors—such as the abused parent’s capacity for care and the resilience of the child—also matter, as do interventions to reduce risk and offer protection. Thus, the whole of the child’s situation needs to be considered and appropriate interventions offered by the case worker in charge of the assessment. 

A systematic review on decision-making within child welfare more generally concluded that structured decision-making enables a more child-centered and holistic approach that takes the child’s family and environment into account [47] (see also [48,114]). However, inter-rater agreement on decisions was not improved, and the authors argue that child welfare and child protection must find additional inspiration from other areas, as research on decision-making processes in child welfare and child protection is still rare. The approach to risk assessment outlined in this manuscript is aligned with commentators arguing that what is needed is step-by-step models for risk assessment where case workers draw on different instruments and methods to assess the risk to partners and children, and then integrate the results into an overall conclusion [23,25]. This is against the backdrop of the complexity of these cases where both adults and children are at risk. A model or support to the how information to cover all of these aspects should be gathered, or how case workers can move from the gathering of information to the analysis and assessment of risk in a short-term as well as long-term perspective, is discussed elsewhere [116,117].

### 4.4. Limitations

The agenda for holistic and child-centered risk assessments when children experience violence in a family setting outlined in this manuscript is based on empirical findings about risks for children, drawn from the research literature. However, it should be recognized that what is discussed is not an instrument, and although work to develop a model for how an assessment based on the different areas of risk can be carried out in practice and studies of the feasibility and usability of this approach in child welfare services are ongoing [116,117], so far this model has not been tested scientifically. What is offered in this manuscript should thus be regarded as a first step in addressing the lack of methods developed for the assessment of risks for children exposed to violence, and a step which needs to be followed by further development and studies. 

## 5. Conclusions

The vast and broad literature on children exposed to violence in a family setting shows that holistic risk assessments placing the individual child at the center should recognize at least four different areas of risk: (1) child safety, i.e., known risk factors for severe and dangerous violence aimed at both adults and children and how they play out in the individual case. Children’s agency needs to be recognized, and in addition to that, (2) the child’s response in situations with violence should be considered. Furthermore, (3) the child’s perspective, especially fear and feelings of powerlessness in situations with violence should be considered, both as children have a right to be heard in all processes that concern them, but also as continued fear and feelings of powerlessness undermine children’s recovery after being subjected to violence. The extensive literature on the long-term negative effects of exposure to violence demonstrates the importance of considering (4) developmental risks, e.g., instability in the child’s situation and care arrangements; lack of a carer/parent as a “secure base” and “safe haven”; the child developing difficulties due to the violence (e.g., PTSD or complex trauma reactions); problems in parents’ caring capacities in relation to a child with experiences of, and reactions to, violence; and lack of opportunities for the child to make sense of, and create meaning in relation to, experiences of violence. In addition, the need for immediate intervention and safety planning in the current situation also needs to be holistic and include dangerousness, the child’s responses, the child’s perspectives, and take developmental risks into account.

## Figures and Tables

**Table 1:** Child Safety.

Study	Type of Study	Country/Countries	Influence on Child Centered Risk Assessment
Abramsky et al., 2011 [45]	Cross sectional study (*n* = 24,097)	Bangladesh, Brazil, Ethiopia, Japan, Namibia, Peru, Republic of Tanzania, Samoa, Serbia and Montenegro, and Thailand.	Risk factors for intimate partner violence (IPV), including victim vunerability.
Almond 2017 [46]	Quantitative study (*n* = 1441) IPV/DV	Great Britain	Criminal history, problems with alcohol, separation and fright reported by the victim was significantly associated with increased risk of IPV/domestic violence (DV).
Bartelink et al., 2015 [47]	Systematic review; 5 reviews and 12 original studies of child maltreatment (CM) methods to improve decision making	Predominantly US	Structured decision making is child centered and holistic and makes the decision process more systematic and transparent.
Bartelink et al., 2017 [48]	Quantitative study; risk assessment tools (RAT) used on CM case vignettes & prospective design in real life cases	Netherlands	Interrater reliability and predictive validity of this consensus-based RAT constructed for child welfare purposes was low to moderate. The importance of combinations of risk factors is discussed.
Belfrage et al., 2012 [49]	Feasibility Study	Sweden	Findings support the potential utility of structured risk assessment and management of IPV.
Campbell 2007 [50]	Narrative Review (35 studies)	Predominately US	Lethal IPV violence. Number one risk for femicide is prior domestic violence
Dababnah et al., 2018 [51]	Systematic Review (11 studies)	Predominately US	Child disability associated with higher risk for child abuse (CA).
Durrant & Ensom 2012 [52]	Narrative review	Predominately North America	Risk factors for CA.
Fang et al., 202 [53]	Systematic review and meta-analysis (98 studies)	Predominately US	Violence against disabled children, including risk factors.
Dubowitz et al., 2011 [54]	Longitudinal study (*n* = 332 families)	US	Risk factors for CA.
Fonagy 1991 [55]	Theoretical article	United Kingdom	Theory of reflective functioning.
Goldman et al., 2003 [56]	Textbook	US	Key issues in interventions against CA.
Graham et al., 2021 [57]	Systematic review; 43 studies of 18 risk assessment tools (RATs) for IPV homicide & IPV re-assault.	Eight countries	Some instruments have been tested for reliability and validity with various results. Research on the feasibility of using these instruments in practice settings is lacking.
Hilton et al., 2010 [58]	Textbook	US	Risk assessment for domestically violent men: Tools for criminal justice, offender intervention, and victim services.
Hilton et al., 2021 [59]	Quantitative study (*n* = 258)	US	RAT study of men charged with IPV against female intimate partners. Relatively higher inter-rater reliability was found on items regarding past assaults, use of weapons but lower agreement for items relating to mental health as well as some victim-related variables.
Hindley et al., 2006 [60]	Systematic review (16 studies)	US	Risk factors for reoccurring CA.
Kropp et al., 2010 [61]	Textbook	Predominantly US and Canada	Perpetrator situation and characteristics, victim vulnerability.
Li et al., 2011 [62]	Longitudinal study (*n* = 405)	US	Risk and protective factors for CA
Messing et al., 2017 [63]	Quantitative (*n* = 254)	US	Validity of lethal, near lethal and severe violence The Danger Assessment instrument. High sensitivity and low specificity.
Millner & Crouch 2018 [64]	Systematic review CA	Not reported	Inconclusive results regarding which combination of risk factors best predicts current or future child physical abuse. Obtaining information from as many sources as possible is however recommended.
Nicholls et al., 2013 [27]	Systematic review; 11 studies of Risk assessment tools (RAT) IPV/DV	Predominantly US	RATs significantly improve accuracy in decisions in IPV/DV cases; risk factors vary with instrument.
Northcott 2008 [26]	Narrative review IPV/DV	Predominantly US & Canada	RATs promote transparency and accountability in IPV/DV cases, accuracy in predicting recidivism varies.
Radford et al., 2006 [25]	Textbook	United Kingdom	Risk assessment in child welfare practice, step by step model.
Shlonsky & Friend, 2007 [23]	Narrative review (previous 30 years)	Predominately US	Critique of actuarial tools as they are constructed for specific purposes and therefore do not include all major risk factors.
Sibert et al., 2002 [65]	Population based and Cross sectional study	United Kingdom	Increased risk for CA in young children, CA a significant problem for children younger than 1 year.
Stith et al., 2009 [11]	Meta-Analysis (155 studies)	Predominantly US.	High risk factors for CA, such as parent anger/hyper-reactivity, family conflict, signs of CA, blaming the child.
US Dep. 2007 [66]	Report	US	Risk factors for CA.
White et al., 2015 [67]	Systematic review (15 studies)	United Kingdom, US	Risk factors for CA.
Wickström et al., 2017 [68]	Register study (*n* = 478,577)	Sweden	Risks of CA associated with parental intellectual disabilities.

**Table 2:** The Child’s Response.

Study/Reference	Type of Study/Data	Countries	Influence on Child Centred Risk Assessment
Edleson et al., 2007 [40]	Review of measures of child exposure to domestic violence (5 instruments)	US	Different child responses in situation with violence.
Edleson et al., 2008 [41]	Validation study (*n* = 65)	US	Different child responses in situations with violence.
Fusco & Fantuzzo 2009 [5]	Cross-sectional study (*n* = 1581)	US	Child involvement to protect; links between child intervention to protect and physical harm to the child.
Howard, D. E. 1999 [70]	Cross-sectional study (*n* = 33)	US	Child involvement to stop violence.
Orford et al., 2005 [71]	Textbook	Australia, United Kingdom, US	Child responding to violence through violence.
Åkerlund 2017 [72]	Qualitative study (*n* = 10)	Sweden	Sibling relationships in the context of DV.
Åkerlund & Sandberg 2016 [73]	Qualitative study (*n* = 10)	Sweden	The role of older children, sibling relationships.
Øverlien 2017 [74]	Qualitative study (*n* = 25)	Norway	The relationship between children’s responses and age.
Øverlien & Hydén 2009 [75]	Qualitative study (*n* = 15)	Sweden	Child responses in situations with violence and afterwards.

**Table 3:** The Child’s Perspective.

Study/Reference	Type of Study/Data	Countries	Influence on Child Centred Risk Assessment
Cohen et al., 2018 [77]	Summary of empirical support for TF-CBT for children	Norway, US	Perpetrator contact as challenge in treatment of child.
Howard et al., 1999 [70]	Cross sectional (*n* = 333)	US	Parents underestimating child exposure and psychosocial functioning.
Hungerford et al., 2010 [76]	Cross sectional (*n* = 75)	US	Parents underestimating child exposure.
Holt et al., 2008 [78]	Narrative review (1995–2006)	Not specified	Child experience of post separation stalking and fear.
Katz et al., 2020 [79]	Qualitative (*n* = 29)	Finland, United Kingdom	Child experience of post separation stalking and fear.
Noble Carr et al., 2020 [80]	Meta-Synthesis (32 studies)	Australia, North America, United Kingdom	Children’s views on DV and impact; feelings of fear and helplessness.
Onsjö et al., 2022 [22]	Qualitative study (*n* = 13)	Sweden	Ongoing violence undermining treatment interventions for children.

**Table 4:** Developmental risks.

Study/Reference	Type of Study/Data	Countries	Influence on Child Centred Risk Assessment
Austin et al., 2019 [81]	In-depth systematic literature review (26 samples)	Predominantly Europe & US	Effect of IPV on women’s parenting ability and behaviours.
Bearss & Eyberg 1998 [82]	Cross sectional study (*n* = 53)	US	Parenting alliance in the marital relationship in general and association with marital adjustment and child behaviour problems.
Cater 2004 [32]	Qualitative study (*n* = 10)	Sweden	Children’s perspectives on father’s violence against mother.
Cater & Forssell 2014 [83]	Qualitative study (*n* = 10)	Sweden	Children’s, whose fathers have subjected their mothers to IPV, perspectives on fathers’ care.
Chan & Yeung 2009 [84]	Meta-analysis (37 samples)	Predominantly Europe & US	Effects of family violence on children’s adjustment.
Chiesa et al., 2018 [85]	Meta-Analytic Study (21 samples)	Predominantly Europe & US	Impact of IPV on victim parenting.
Delvecchio et al., 2015 [86]	Cross sectional study (*n* = 1606)	Italy	Co-parenting alliances as mediator on the influence of parents’ trait anxiety on family system maladjustment and parenting stress.
DeVoe & Smith 2002 [87]	Qualitative study (*n* = 43)	USA	Diversity in mothers’, to children exposed to IPV, descriptions of how their own experiences of victimization has affected their caring behaviours.
Eskonen 2005 [33]	Qualitative study (*n* = 7)	Finland	Children’s, exposed to DV, narratives of violence.
Evans et al., 2008 [88]	Meta-Analytic study (60 samples; *n* = 7602)	Predominantly Europe & US	The relationship between childhood exposure to DV domestic violence and their internalizing, externalizing, and trauma symptoms.
Evans, et al., 2022 [89]	Meta-Analytic Study (13 samples)	Predominantly Europe & US	Children’s, exposed to IPV, acceptance and appraisals of IPV.
Fainsilber & Low 2004 [90]	Cross sectional study (*n* = 130)	US	Relations between DV, coparenting, family-level processes and children’s adjustment.
Fearon, R. P., et al., 2010 [91]	Meta-Analytic Study (69 samples; *n* = 5947)	Predominantly Europe & US	Relation between insecure and disorganized attachments and externalizing problems.
Graham-Bermann et al., 2007 [92]	Efficacy trial (*n* = 181)	US	Reltaionship between exposure to IPV andattitdes to violence.
Groh et al., 2012 [93]	Meta-Analytic Study (42 samples; *n* = 4614)	Predominantly Europe & US	Relation between insecure and disorganized attachments and internalizing symptoms.
Hackett 2014 [94]	Textbook	Great Britain	General aspects of parenting capacity and child development.
Horwath & Platt 2019 [95]	Textbook	Great Britain	General aspects of assessing vulnerable children
Hultmann et al., 2022 [18]	Cross sectional study (*n* =578)	Sweden	Association between exposure to IPV or CA (single) and exposure to IPV and CA (double) and psychiatric symptoms and post trauma impact.
Humphreys & Stanley 2006 [96]	Textbook	Great Britain	Exposure to IPV as a risk of undermining the child-abused parent relationship.
Kitzmann et al., 2003 [14]	Meta-Analytic Study (118 samples)	Predominantly Europe & US	Association between exposure to IPV and psychosocial outcomes.
Kobak & Madsen 2008 [97]	Textbook	US	General aspects of the carer’s capacity to provide protection, comfort, and help the child with emotional regulation.
Konold & Abidin 2001 [98]	Multigroup confirmatory factor analysis (*n* = 1224)	US	General aspects of parenting alliance.
Leira 2002 [99]	Textbook	Nordic countries	Violence as surrounded by taboo, making it difficult to talk and make sense about.
Letourneau et al., 2007 [100]	Longitudinal study (*n* = 3245)	Canada	Relation between exposure to family violence and parenting behaviors.
Levendosky & Graham-Bermann 2003 [101]	Observational study (*n* = 103)	US	Association between DV and parenting behaviour.
Levendosky et al., 2018 [102]	Textbook	US	Association between DV and the Maternal–Child relationship and child functioning.
McCloskey & Walker 2000 [103]	Cross sectional study (*n* =337)	US	Association between PTSD & DV.
Miller et al., 2014 [13]	Cross sectional study (*n* =703)	Sweden	Associations between exposure to IPV in childhood and adult mental health.
McGee 2000 [34]	Textbook	Great Britain	Children’s experiences of being exposed to DV.
Mullender 2004 [104]	Textbook	Great Britain	Providing support to children exposed to DV.
Mullender et al., 2002 [35]	Textbook	Great Britain	Children’s perspectives on DV
Parkinson & Humphreys 1998 [105]	Conceptual article	Australia & Great Britain	Children’s perspectives on DVand implication for child protection.
Peled & Barak-Gil 2011 [106]	Qualitative study (*n* = 10)	Israel	Perception of mothering among mothers subjected to DV.
Saltzman et al., 2005 [107]	Cross sectional, comparison (*n* = 48)	US	Associations between exposure to IPV an child psychological and physiological functioning.
Sameroff 2009 [43]	Textbook	US	A developmental psychopathology perspective on child development in general.
Sousa et al., 2011 [108]	Longitudinal study (*n* = 457)	US	Unique and combined effects of child abuse and children’s exposure to domestic violence on later attachment to parents and antisocial behaviour.
Stover 2013 [109]	Initial feasibility study (*n* =10)	US	IPV and aspects of co-parenting.
Wolfe et al., 2003 [31]	Meta-Analytic Study (41 samples, *n* = 5088)	Predominantly Europe & US	Associations between children’s exposure to DV and social, emotional, behavioural, cognitive, and general health functioning.

## Data Availability

Not applicable.
